# Laser Ablation-Assisted Synthesis of Plasmonic Si@Au Core-Satellite Nanocomposites for Biomedical Applications

**DOI:** 10.3390/nano11030592

**Published:** 2021-02-26

**Authors:** Ahmed Al-Kattan, Gleb Tselikov, Khaled Metwally, Anton A. Popov, Serge Mensah, Andrei V. Kabashin

**Affiliations:** 1 Aix-Marseille University, CNRS, LP3, Campus de Luminy, 13013 Marseille, France; gleb.tselikov@univ-amu.fr (G.T.); a.popov.fizteh@gmail.com (A.A.P.); 2 Moscow Institute of Physics and Technology, Center for Photonics and 2D Materials, 141700 Dolgoprudny, Russia; 3 Aix-Marseille University, CNRS, LMA, 13013 Marseille, France; mensah@lma.cnrs-mrs.fr (K.M.); metwally@fresnel.fr (S.M.); 4 Aix-Marseille University, CNRS, Institut Fresnel, Centrale Marseille, 13013 Marseille, France; 5 MEPhI, Bio-Nanophotonics Laboratory, Institute of Engineering Physics for Biomedicine (PhysBio), 31 Kashirskoe sh., 115409 Moscow, Russia

**Keywords:** pulsed laser ablation in liquids, Si@Au core-satellite, core-shell, plasmonic nanoparticles, Mie theory, biomedical applications

## Abstract

Owing to strong plasmonic absorption and excellent biocompatibility, gold nanostructures are among best candidates for photoacoustic bioimaging and photothermal therapy, but such applications require ultrapure Au-based nanoformulations of complex geometry (core-shells, nanorods) in order to shift the absorption band toward the region of relative tissue transparency (650–1000 nm). Here, we present a methodology for the fabrication of Si@Au core-satellite nanostructures, comprising of a Si core covered with small Au nanoparticles (NP), based on laser ablative synthesis of Si and Au NPs in water/ethanol solutions, followed by a chemical modification of the Si NPs by 3-aminopropyltrimethoxysilane (APTMS) and their subsequent decoration by the Au NPs. We show that the formed core-satellites have a red-shifted plasmonic absorption feature compared to that of pure Au NPs (520 nm), with the position of the peak depending on APTMS amount, water−ethanol solvent percentage and Si−Au volume ratio. As an example, even relatively small 40-nm core-satellites (34 nm Si core + 4 nm Au shell) provided a much red shifted peak centered around 610 nm and having a large tail over 700 nm. The generation of the plasmonic peak is confirmed by modeling of Si@Au core-shells of relevant parameters via Mie theory. Being relatively small and exempt of any toxic impurity due to ultraclean laser synthesis, the Si@Au core-satellites promise a major advancement of imaging and phototherapy modalities based on plasmonic properties of nanomaterials.

## 1. Introduction

Based on excitations of oscillations of free electrons (plasmons), Au-based nanomaterials can provide strong resonant absorption opening access to the development of novel photonic imaging and therapy modalities [1]. However, these modalities are difficult to implement using simple spherical Au nanoparticles (NP), as the plasmonic absorption band of these NPs is centered around 520−560 nm, which is out of the optical window of relative tissue transparency (650−1000 nm). To solve such a plasmonic mismatch problem, one has to design complex Au-based nanoarchitectures such as elongated Au nanorods [2] or dielectric-Au core-shell NPs [3,4], which are capable of red-tuning the plasmonic absorption toward the transparency window. In the nanorods, the position of such plasmonic feature is controlled by the aspect ratio of nanorod axes, while in the case of the core-shells it can be tuned by varying refractive index of the core and the ratio of their size/thickness. 

The engineered nanorods and core-shells have already demonstrated high efficiency as sensitizers of light-induced hyperthermia cancer therapy [2,3,4] and as contrast agents in optical and photoacoustic imaging [5,6,7,8], but the employment of these nanostructures is not free of problems and challenges. One of the main problems is related to residual toxicity of these nanostructures arising due to their contamination by by-products during conventional chemical synthesis. Indeed, the nanorods are stabilized by non-biocompatible cetyltrimethylammonium bromide [9], while chemical synthesis of SiO_2_@Au core-shell NPs typically requires the involvement of hazardous raw products such as chloride, citrate, nitrate, and other organic solvents under severe acidic or alkaline pH conditions [3,4,10]. In addition, the elongated shape of the nanorods can complicate their transport in the organism [11], while only relatively large (>100–150 nm) SiO_2_@Au core-shells can provide absorption in the tissue transparency window, which complicates their excretion from the organism [12]. The required red-shift of the plasmonic peak could be reached at smaller core-shell sizes if a high refractive index material such as silicon (Si) is used a core instead of silica (SiO_2_) [13,14], but the fabrication of these structures by chemical methods is typically very complicated. 

Pulsed laser ablation in liquids presents a physical alternative, which can offer a solution of the problem of purity of synthesized nanomaterials [15,16]. This approach relies on laser radiation-caused ablation of material from a solid target to initiate the generation of nanoclusters [17], which can then be released either into a gaseous medium to form a substrate-supported nanostructured film or a powder [18,19], or into a liquid medium to form a solution of NPs [20,21,22]. In all cases, the ablation can be performed in ultrapure environment (inert gas, deionized water, etc.) to exclude any contamination of the nanoparticle surface by side-products or stabilizing ligands, which is critically important for projected biomedical applications in vitro and in vivo [23,24]. As an example, we recently elaborated the technique of femtosecond (fs) laser ablation in water and organic media, which renders possible an efficient control of size characteristics of NPs from a variety of materials, including Au [22,25], Si [26,27], TiN [28,29] NPs. It is important that the laser-ablative technique can offer opportunities for the synthesis of complex structures such as core-shells. Such structures can be formed spontaneously during the ablation or co-ablation of materials having appropriate physico-chemical properties [30,31,32,33,34], or by the combination of laser ablation with an additional chemical reduction step using HAuCl_4_ solutions [35,36]. In particular, the latter approach was used for the fabrication of plasmonic SiO_2_@Au [35] and Si@Au [36] core-shell NPs. However, the first laser-ablative approach typically leads to a wide size and shape dispersion of formed composites, while the second combination approach still cannot avoid residual contamination by side products of the chemical reduction process.

This work is devoted to the search of alternative laser-assisted methodologies for the synthesis of contamination-free Au-based nanostructures having plasmonic feature in the region of relative tissue transparency. We describe a methodology for the synthesis of Si@Au core-satellite nanocomposites comprising of by relatively large Si core decorated by small Au NPs, which is based uniquely on bare laser-synthesized Si and Au NPs, and does not involve the chemical reduction step. In this methodology, the NPs are modified by organosilane APTMS molecules and then assembled into complex core-satellite structures. We show that such a geometry makes possible the generation of plasmonic absorption feature in the tissue transparency window even for relatively small sizes of core-satellite constituents (less than 50 nm). Optical properties of Si@Au core-satellite nanocomposites are consistent with calculations based on multi-layer Mie-theory modelization. Combined with a high purity of products involved in the assembling of the core-satellites, the synthesized structures look very promising for biomedical applications. 

## 2. Materials and Methods

### 2.1. Fabrication of Si@AuNPs:

#### 2.1.1. Laser Synthesis of Bare Si and Au NPs:

Solutions of colloidal Si NPs were obtained from 0.5 µm Si micropowder, which was preliminary prepared by mechanical milling of a Si wafer as it was described in [26,27]. Si powder was dispersed by a sonication for 15 min in a solution of 4.5 mL of water−ethanol at two concentrations of 0.2 mg.mL^−1^ and 0.4 mg.mL^−1^. In total, three ratios of 100−0, 50−50 and 0−100 of water−ethanol were tested. Si micropowder solutions were then fragmented for 60 min using radiation from a Yb:KGW femtosecond laser (Amplitude systems, France, wavelength 1025 nm, repetition rate 10 kHz, pulse duration 480 fs). The laser beam was focused in the center of a cuvette filled by micropowder solutions using a 75 mm lens. The homogenization of the solution was done by a magnetic stirrer. Unfragmented Si powder was removed by the centrifugation at 3500 rpm.min^−1^ for 20 min.

Au NPs were synthesized using the same laser in the ablation geometry, as previously described in [22,37]. For this, a gold target (99.99%, Sigma Aldrich, Saint-Quentin Fallavier, France) immersed in 7 mL of aqueous 0.1% NaCl solution was irradiated by a focused laser beam. To ensure a homogenous ablation process, the ablation target was continuously moved with 2 mm/s speed using motorized linear translational stages (Newport, Every, France).

#### 2.1.2. Modification of Si NPs:

The silanization step was performed by adding a solution of 3-aminopropyltrimethoxysilane (APTMS) (Sigma Aldrich, Saint-Quentin Fallavier, France) to Si NPs solutions at ambient condition. The obtained mixture was stirred at room temperature for 24 h. In total, three volumes of 1 mL, 0.5 mL and 80 µL of APTMS were tested to find the optimized condition of silanization.

#### 2.1.3. Formation of Si@Au NPs:

Aqueous solutions of Au NPs were gently added to Si-APTMS NPs solutions at different volume ratios (1:1, 1:10, 1:30 of Si:Au NPs solutions) and stirred at ambient conditions for 24 h.

#### 2.1.4. Physicochemical Characterizations:

A high-resolution transmission electron microscopy (HR-TEM) system (JEOL JEM 3010, Milpitas, USA) operating at 300 kV was employed to perform structural characterizations (size distribution, crystalline structure, morphology) of Si@Au nanostructures. Samples for the electron imaging were prepared by placing a drop of a NPs solution on a carbon coated copper TEM grid (200 mesh, Oxford instruments, Abingdon-on-Thames, UK) and its drying at ambient conditions. Statistical data were obtained based on analysis of size characteristics from several hundreds of NPs.

ζ-potential and dynamic light scattering (DLS) measurements were performed using a Zetasizer Nano ZS system (Malvern Instruments, Palaiseau, France).

Optical extinction spectra of NPs were measured by a UV−Vis spectrophotometer (UV-2600, Shimadzu, Marne-la-Vallée, France) using 10 mm optical path length quartz cuvettes.

### 2.2. Optical Simulation: Mie Theory Modeling

The objective of this simulation is to predict the absorption efficiency of multilayered core-shell NPs. Absorption and scattering of spherical NP can be described analytically using Mie theory taking into account NP size and optical properties of material, as previously demonstrated in [38]. This model has been extended to describe multilayer or coated particles, as well as be generalized to find a solution for scattering by more than one particle in a field, as explained in [39]. Meunier’s group implemented a “matriochka” model using an open access code (Matlab) in order to optimize material, size, and shape of a NPs for nanocavitation applications [40]. On the other hand, Plain’s group studied the core−shell size ratio versus the variations of the absorption efficiency [14]. In this work, optical spectra effective cross-sections ($\sigma_{abs}:$ absorption, $\sigma_{sca}:$ scattering, and $\sigma_{ext}:$ extinction) were modeled by calculating the optical response of a multilayered particle (Si, Au and APTMS) with homogeneous fine-layers of a finite thickness. Absorption and scattering efficiencies present the ratio between effective and geometrical cross-sections ($Q_{abs}=\frac{\sigma_{abs}}{\pi b^{2}}$, $Q_{sca}=\frac{\sigma_{sca}}{\pi b^{2}}$).

When the mean free path of electrons (MFP) is comparable with the shell thickness or to the particle diameter (a few nanometers), it is important to take into account the size-dependency of the dielectric function [14,41]. In this case, scattering of conduction band electrons on the particle surface results in a reduced effective mean free path ($L_{eff}$) and the increase in the resonant linewidth of plasmon polariton (*Γ*) [42,43]. Mathiessen’s rule quantifies this effect through the relation: $\Gamma =\Gamma_{bulk}+\frac{V_{F}}{L_{eff}}$ leading to a modified dielectric function $\epsilon \left(L_{eff},\omega \right)$ [44,45]:

(1) $$\epsilon \left(L_{eff},\omega \right)=\epsilon {\left(\omega \right)}_{Au}+\frac{\omega_{p}^{2}}{\omega^{2}+i\omega \Gamma_{bulk}}-\frac{\omega_{p}^{2}}{\omega^{2}+i\omega \left(\Gamma_{bulk}+\frac{V_{F}}{L_{eff}}\right)}$$

where $\omega_{p}=1.37\times 10^{16}\;s^{-1}$ is the plasma frequency, $V_{F}$ is the Fermi velocity of conduction electrons (for Au, $V_{F}=1.4\times 10^{6}$ m/s), and $\Gamma_{bulk}=\frac{V_{F}}{L_{\infty }}$ is the plasmon polariton resonant linewidth for bulk material (for Au, Drude MFP $L_{\infty }=42\;nm$). The ratio $\frac{V_{F}}{L_{eff}}$ may be classically interpreted as the effective rate of scattering of conduction band electrons on the particle surface. In other words, the surface scattering can reduce the absorption efficiency and broaden the plasmon resonance.

The shell geometry also affects the surface scattering. For a spherical shell geometry within the linearized thin shell limit, at which $L_{eff\;}$≈ twice the shell thickness, the billiard’s expression of MFP is written as [43]:

(2) $$L_{eff}=\frac{4\left(r_{2}^{3}-r_{1}^{3}\right)}{3\left(r_{2}^{2}+r_{1}^{2}\right)}$$

where $r_{1}$ and $r_{2}$ are radii of the inner and outer surfaces of the shell, respectively. The relations (2) and (1) were implemented in to take in account the effect of size and shape of fine-layered spherical NP.

## 3. Results and Discussions

**Figure 1:**
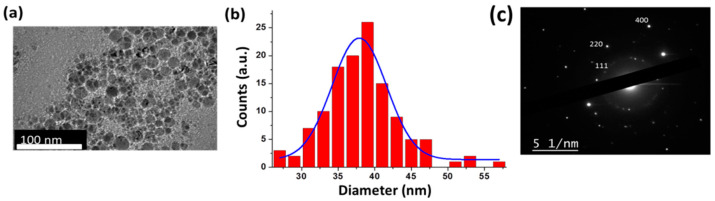
Typical HR-TEM image of Si NPs prepared by laser ablation in water (**a**) and a corresponding size distribution (**b**). Electron diffraction patterns of Si NPs (**c**).

A typical HR-TEM image of laser-synthesized Si NPs with corresponding statistical size analysis and electron diffraction pattern is shown in Figure 1. As shown in the figure, laser-synthesized Si NPs had the characteristic spherical shape, while the statistical analysis reveals the mean size of about 37 ± 3 nm. As follows from the electron diffraction pattern (Figure 1c) and a corresponding lattice constant table (Table 1), the formed nanoparticles were crystalline, which is confirmed by the presence of diffraction rings, corresponding to (111), (220), and (400) crystalline planes of cubic diamond structure.

**Figure 2:**
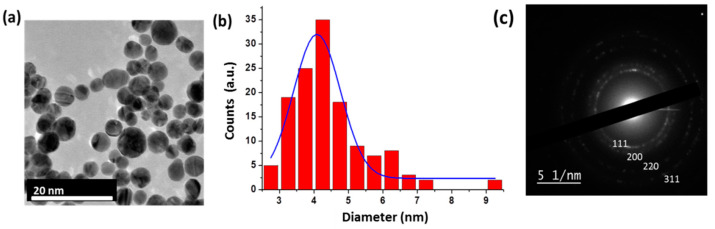
Typical HR-TEM images of Au NPs (**a**) prepared by laser ablation process in water, with corresponding size distribution (**b**) Electron diffraction pattern of Au NPs with the corresponding interplanar spacing d*_hkl_* (**c**).

Au NPs prepared by laser ablation in aqueous 0.1% NaCl solutions also had a spherical shape (Figure 2a), while the mean size was about 4 nm ± 0.8 nm (Figure 2b). As follows from the electron diffraction pattern (Figure 2c) and a corresponding lattice constant table (Table 2), the formed NPs had an Au FCC lattice.

Despite relatively high reactivity of surfaces of Si and Au NPs, the oxidation states of these surface makes the interaction of Si Au NPs difficult due to electrostatic repulsion effect between the oxidized species of SiO^2−^ and AuO^−^ exhibiting negative surface charges of −45 ± 1.5 mV and −32 ± 2.6 mV, respectively. In order to couple the NPs in one nanoformulation, the surface of Si NPs was activated by an organosilane molecule 3-aminopropyltiethoxysilane (APTMS), which is one of widely used ligands to coat inorganic NPs and functionalize them with a variety of molecules. Such a molecule exhibits two alkoxy and amine functional groups on chemical chain extremities, which can make the coupling process between Si NPs and Au NPs possible [46]. As suggested in the literature, a possible chemical link can be due to hydrolysis of methoxy groups of APTMS, which react with the hydroxyl Si-OH group on the oxidized Si NPs surface to form Si-O-Si bonds. In this case, a protonated amino host of Si NPs is formed, which is capable of reacting with the oxidized surface of Au NPs via electrostatic interactions [46].

To achieve the coupling, we optimized different physicochemical parameters, including APTMS amount and solvent type (e.g., aqueous, ethanol) to prevent any parasitic reaction such as the polymerization of APTMS. To select the optimal extent of silanization of Si NPs, three different volumes (1, 0.5 mL, and 80 µL) of APTMS were mixed with 4.5 mL of fresh colloidal Si NPs solutions, as summarized in Table 3.

**Figure 3:**
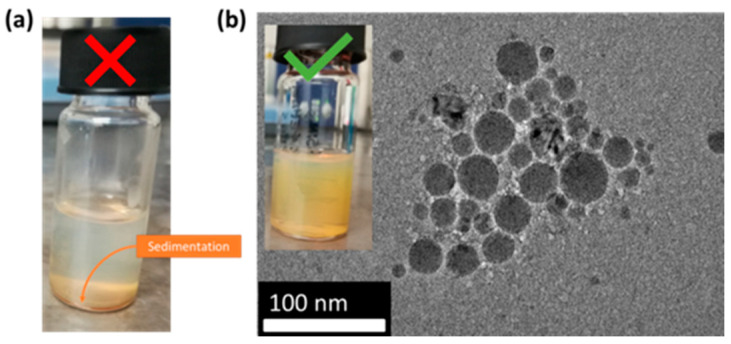
(**a**) Illustration of sedimentation of Si NPs treated with 1 mL of APTMS in 4.5 mL of pure water. (**b**) A stable Si NPs solution treated with 80 µL of APTMS in pure water and corresponding HR-TEM image of Si NPs.

After 24 h of stirring at ambient conditions, Si NPs solutions mixed with 1 and 0.5 mL of APTMS experienced a jellification process, leading to a partial or complete sedimentation of Si NPs in the solution after 3 days (Figure 3a). The observed jellification process was probably due to an excess of APTMS amount, which resulted in a self-condensation and generation of intramolecular hydrogen bonds between APTMS molecules, yielding the formation of poly(aminopropyl)siloxane polymers surrounding the Si NPs [46]. On the other hand, Si NPs solutions mixed with 80 µL APTMS were stable without any change of size characteristics of Si NPs, as confirmed by HR-TEM analysis (Figure 3). Moreover, zeta potential studies of Si NPs solutions, purified from unreacted APTMS, confirmed the increase in surface charge by +2.58 ± 0.20 mV, which was probably due to the presence of NH_3_^+^ groups of APTMS on Si NPs surface. 

**Figure 4:**
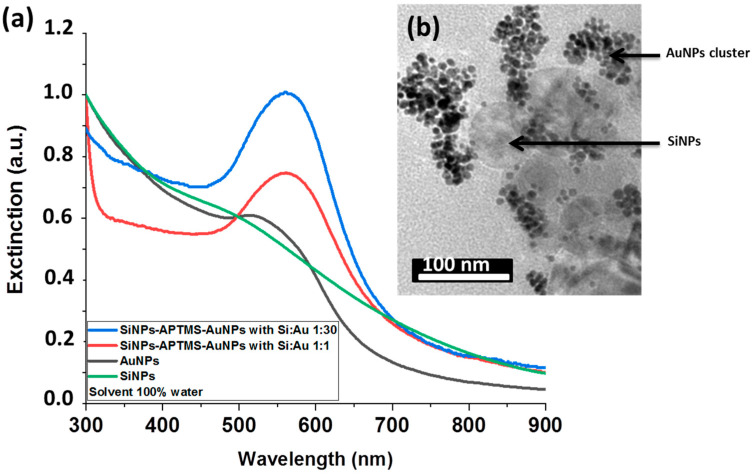
(**a**) UV−Vis spectrum of Si NPs treated with 80 µL of APTMS in pure water solution and then mixed with Au NPs solutions at different ratios (**b**).

Aqueous solutions of Au NPs were then added at increased volume ratios of 1:1 and 1:30 related to Si NPs content into a Si NPs solution treated with 80 µL APTMS for 1 h. The suspension of Si NPs was then rinsed several times by pure water to eliminate unreacted products. Absorbance of so formed Si@Au conjugate was compared to that of pure Si NPs (green curve) and Au NPs (black curve), which were considered as references. As shown in Figure 4a, independently of the volume ratio of Si NPs−Au NPs (1:1 or 1:30 volume ratios), the extinction peak of the Si@Au conjugate was red-shifted by almost 47 nm compared with the initial plasmonic peak of pure Au NPs to reach the maximum at 560−570 nm, suggesting the APTMS-mediated attachment of Au NPs onto the surface of Si NPs (red and blue curves). This conclusion is confirmed by typical HR-TEM images of samples prepared at the ratio of 1:30 (Figure 4b). However, as followed from our observations, Si NPs were only partially grafted by Au NPs, while a significant number of separated Au NPs and their agglomerations were still present in the solution, suggesting a partial silanization effect. It should be noted that aqueous media are typically used with APTMS in order to ensure an efficient hydrolysis of methoxy group of APTMS [46]. However, such a reaction can also lead to a self-condensation of APTMS, which decreases its ability to react with Si-OH of Si NPs. 

**Figure 5:**
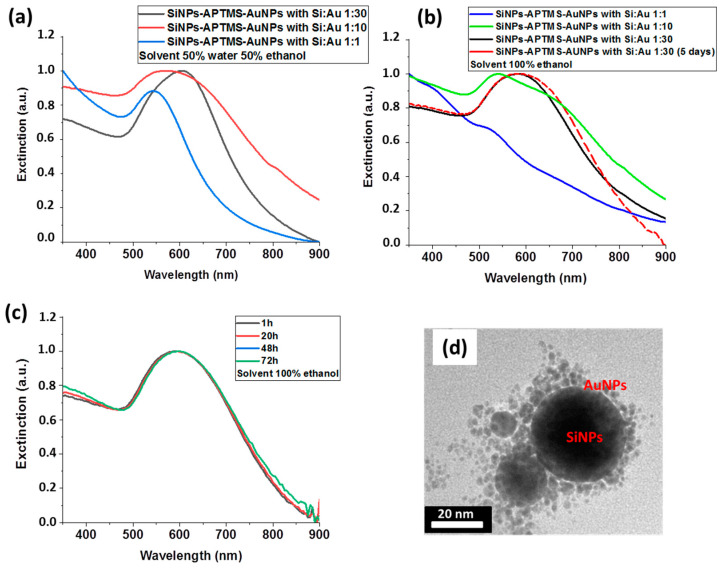
Extinction spectra of Si@Au core-satellite nanocomposites having different Si−Au volume ratio prepared at 50−50 (**a**) and 0−100 (**b**) water−ethanol solutions. (**c**) Extinction spectra after different times of their aging. (**d**) Typical HR-TEM image of Si@Au nanocomposites prepared in 80 µL of APTMS in pure ethanol.

To increase the silanization efficiency and limit self-condensation effects, solutions of ethanol were introduced into the Si NPs solutions at the water−ethanol ratio of 50−50 and 0−100 (Table 3). Then, 80 µL of APTMS was added into colloidal Si NPs suspensions and left for 24 h. Au NPs were introduced at different Si NPs−Au NPs volume ratios of 1−1, 1−10, 1−30 and stirred for 24 h, while one solution prepared at 1:30 was stirred for 5 days. All solutions were rinsed by 50−50 and 0−100 water−ethanol solutions to remove unreacted products. As shown in Figure 5, at a low volume ratio of Au NPs, Si@Au conjugates exhibited similar plasmonic behavior with a characteristic 47 nm red-shift of extinction peak compared to pure Au NPs. However, the increase in the volume ratio of Au NPs up to 1−30 caused a larger red-shift of the extinction peak to reach a maximum of absorbance at 600 nm, while the storage of the samples for 5 days led to its further shift up to 610 nm. The peak maximum was probably broadened due to relative polydispersity of size of nanostructures in the colloidal suspension. 

As shown in Figure 5d, the formed nanostructures had core-satellite geometry, comprising of a large Si core decorated by small Au NPs. In contrast to the nanostructures produced in pure water (Figure 4b), the Si core of Si@Au composites was fully coated with a continuous shell of randomly distributed Au NPs, while separated Au NPs were almost absent in the solution (Figure 5d). Statistical analysis of the size of nanostructures showed that the mean size of silicon core was 37 nm silicon, while the thickness of Au NPs-based shell was about 4 nm, which matched the original size of Si and Au nanoparticle constituents. We also observed that in some cases a second monolayer of Au NPs was formed over the first one, which can be explained by the involvement of different reactive groups of APTMS. Here, we suppose that NH_3_^+^ group of APTMS was linked to Au NPs shell, while Si(OH)_3_ oriented toward the solution was hydrolyzed and provided another free APTMS group to form the second layer of Au NPs. Finally, we assessed the extinction feature different times after running the chemical conjugation protocol. As shown in Figure 5c, Si@Au core-satellites prepared in ethanol at the ratio 1:30 of Si NPs−Au NPs did not demonstrate any alteration of optical feature centered around 600 nm after 72 h.

**Figure 6:**
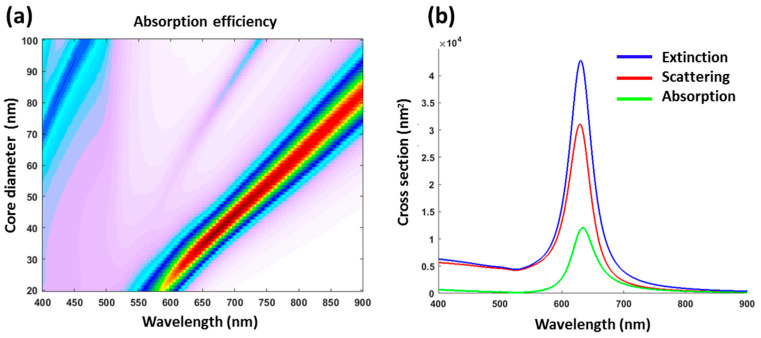
Analytical modeling of absorption of Si@Au NPs prepared in ethanol solvent with 80 µL of APTMS at the ratio of 1−30 of Si NPs−Au NPs based on Mie-theory model (**a**) with corresponding spectra of extinction, scattering and absorption (**b**).

To explain optical properties of formed Si@Au core-satellites, we carried out theoretical simulations using multi-layer Mie-theory calculations. The core-satellites were modeled as multi-layer structures, composed of a Si nanoparticle core surrounded by a 1 nm monolayer of APTMS and a monolayer shell of Au NPs. Refractive index of APTMS was estimated as 1.44 according to literature data. In our simulations, Si-core size was varied from 20 nm to 100 nm, while the thickness of Au NPs-based shell was fixed as 4 nm in accordance with HR-TEM observations (Figure 5d). For simplicity, Au NPs-based shell was assumed to cover the whole core surface, while ethanol was considered as an ambient medium. As shown in Figure 6a, the calculations were in fairly good agreement with experiments. Indeed, calculations for the maximum of plasmonic peak for core-shells provided 640 nm, which was not far from experimentally measured value around 600 nm. A certain discrepancy of theoretical and experimental results was obviously due to simplified model, in which Au shell was considered as continuous layer instead of randomly distributed Au NPs. The presence of an oxide layer on silicon NPs surface could be another reason of the blue shift of experimentally measured extinction spectra compared to results of calculations. As follows from our calculations, for experimental parameters, extinction properties of Si@Au core-satellites are characterized by a strong absorption (Figure 6b), which is important for projected phototherapy applications. The calculations also show that the resonance feature can be tuned dramatically toward the NIR region while the Si-core size increases up to 60 nm. In general, our data confirm a great potential of Si@Au core-satellites for light-induced hyperthermia of cancer.

Thus, we demonstrated a successful fabrication of core-satellite Si@Au nanostructures by chemical assembling of laser synthesized nanomaterials. We believe that the formed nanoformulations can be of importance for potential biomedical applications, including photothermal therapy and photoacoustic imaging. As one of main advantages for such applications, we see exceptional purity of formed nanoformulations due to a complete avoidance of the chemical reduction step. Indeed, Si and Au NPs are produced separately by femtosecond laser ablation in deionized water, which leads to the formation of bare (ligand-free) surface in the absence of any contamination by toxic products. Laser-synthesized Au NPs present a highly safe object for biological systems, as we showed in previous tests in vitro and in vivo [47], although relatively large nanoparticles could have some residual accumulation in organs. On the other hand, laser-synthesized Si NPs are not only safe, but also completely biodegradable as in biological environment they decay into orthosilicic acid and excreted from the body with the urine [26,27], while the dissolution rate of these NPs can be varied from several days to several weeks by changing parameters of the preparation procedure [26]. A subsequent application of chemical protocol on the basis on APTMS is not supposed to add any toxicity as APTMS is a highly biocompatible product with a successful track of applications in biological systems [46]. Another expectation is related to possible gradual decay of core-satellites under long-term exposition to biological environment and a complete excretion of decay products from the organism. In this case, Si NPs are supposed dissolve in physiological conditions independently of their original size [26], while Au NPs are expected to easily excrete from the body as their size appears to be within renal glomerular filtration range (<8 nm) [11]. The study of decomposition properties and biological assessment are now in progress.

The observed strong extinction of core-satellites due to the presence of Au NPs promises successful biomedical applications of Si@Au core-satellite nanocomposites in light-induced hyperthermia of cancer. Although the very peak of plasmonic feature (610 nm) is out of the window of relative tissue transparency, its long tail matches this window and extends over 700–800 nm. We believe that the core-satellite geometry of Si@Au nanocomposites can be advantageous for these applications compared to classical SiO_2_@Au core-shell nanostructures [3,4,5,6] due to a much smaller size. Indeed, the diameter of core-satellites is less than 40 nm, which is in the range of relative safety of nanomaterials for biological systems and should favor transport of nanostructures in vivo reach affected organs and their easier removal from the organism. In addition, the inclusion of laser-ablated nanosilicon in their composition can additionally provide a series of novel theranostic functionalities based on unique intrinsic physico-chemical properties of this material, including the generation of non-linear response for bioimaging [48], hyperthermia-based therapies under radiofrequency excitation [49]. As another potential application, we see the use of core-satellites as probes for Surface Enhanced Raman Scattering (SERS) for the identification of trace amounts of biological species. We already reported the fabrication inverted geometry of Au@Si core-shells, composed of Au core covered by Si shell, with variable contributions of Au and Si constituents [50]. Exhibiting local electric field enhancement due to the excitation of plasmons around 520 nm, such core-shells demonstrated a high efficiency in the identification of bacteria cultures [50]. We expect that Si@Au core-satellites can provide similar enhancement and be used with red-shifted pumping wavelength (e.g., 630, 785 nm) for SERS excitation, which is preferable under some conditions. It should be finally noted that silicon and gold architecture also presents a very promising platform for surface plasmon resonance biosensing [51,52], which can have advantages over classical a glass and gold platform [53,54,55] due to essentially different conditions of plasmon coupling. We believe that the proposed core-satellites can be also explored as nanoscale implementations of such biosensors.

## 4. Conclusions

In summary, we demonstrated a laser-assisted methodology for the fabrication of Si@Au core-satellite nanostructures, comprising of a Si core covered with small Au nanoparticles (NP). The methodology employed laser ablative synthesis of Si and Au NPs in water−ethanol solutions, followed by their chemical modification and linking via organsilane APTMS molecules. Several parameters such as APTMS concentration and type of synthesis medium were optimized to obtain stable Si@Au nanocomposites having a relatively small size (less than 40 nm). The core-satellites had a strong plasmonic peak centered around 600–610 nm with a long tail over 700–800 nm, which is red-shifted compared to spherical Au NPs and overlaps with biological transparency window. The generation of this peak was consistent with results of theoretical modeling using Mie theory. Being relatively small compared to commercial silica-gold nanoshells and exempt of any toxic impurity due to ultraclean laser synthesis, the Si@Au core-satellites promise the advancement of plasmonic modalities for cancer theranostics.

## Figures and Tables

**Table 1:** Interplanar spacing d*_hkl_* for observed crystalline planes of Si NPs.

Ring	d_hkl_ (Å)	hkl	a (Å)	Space Group
1	3.13	111	5.42	Cubic diamond Fd3m
2	1.91	220	5.40	Cubic diamond Fd3m
3	1.37	400	5.47	Cubic diamond Fd3m

**Table 2:** Interplanar spacing d*_hkl_* for observed crystalline planes of Au NPs.

Ring	d_hkl_ (Å)	hkl	a (Å)	Space Group
1	2.38	111	4.08	Cubic fcc Fm3m
2	2.04	200	4.08	Cubic fcc Fm3m
3	1.46	420	4.08	Cubic fcc Fm3m
4	1.22	311	4.08	Cubic fcc Fm3m

**Table 3:** Variable volumes of APTMS used for the silanization of Si NPs prepared by laser ablation at three different water/ethanol ratios.

APTMS Volume	Ratio of Water to Ethanol		
	100:0	50:50	0:100
1 mL	Jellification/Complete sedimentation	_	_
0.5 mL	Jellification/Partial sedimentation	_	_
80 µL	Stable	Stable	Stable
